# *Aspergillus fumigatus* carrying TR34/L98H resistance allele causing complicated suppurative otitis media in Tanzania: Call for improved diagnosis of fungi in sub-Saharan Africa

**DOI:** 10.1186/s12879-016-1796-4

**Published:** 2016-09-02

**Authors:** Martha F. Mushi, Gustave Buname, Oliver Bader, Uwe Groß, Stephen E. Mshana

**Affiliations:** 1 Department of Microbiology and Immunology, Weill Bugando School of Medicine, Catholic University of Heath and Allied Sciences (CUHAS), P.O. BOX 1464, Mwanza, Tanzania; 2 Department of Surgery, Bugando Medical Center, Mwanza, Tanzania; 3 Institute of Medical Microbiology, University Medical Center Goettingen, Göttingen, Germany

**Keywords:** Suppurative otitis media, Moulds, Yeast, Diagnosis of fungi

## Abstract

**Background:**

Suppurative otitis media (SOM) is a major public health concern worldwide and is associated with increased morbidity. Cases of fungal suppurative otitis media were studied to establish the effect of fungi in otitis media.

**Methods:**

Ear swabs from 410 patients were collected aseptically using sterile cotton swabs from discharging ear through perforated tympanic membrane. Swabs were subjected to microscopic and culture investigations. The species of fungal growing on Sabouraud’s agar were identified using MALDI-TOF MS. For moulds broth micro dilution method following EUCAST guidelines was employed to determine susceptibility patterns against itraconazole, voriconazole and posaconazole.

**Results:**

A total of 44 (10.74 %) cases with positive fungal culture growth were studied. The median age of patients with fungal infection was 29.5 (IQR 16–43) years. Of 44 patients; 35 (79.6 %) had pure growth of one type of fungal. *Candida albicans* was the most common fungus isolated (*n* = 13; 29.6 %) followed by *Aspergillus versicolor* (*n* = 8; 18.2 %). A total of 7 (15.9 %) patients had disease complication at time of enrollment; of them 6 (13.6 %) had hearing loss. On follow up 7 (15.9 %) had poor treatment outcome. All five *Aspergillus fumigatus* strains resistant itraconazole with reduced susceptibility to voriconazole and posaconazole carried carrying TR34/L98H resistance allele. In addition, all *Penicillium citrinum* isolates were resistant to voriconazole while all *Penicillium sumatrense* were resistant to both itraconazole and voriconazole. There were non-significant association of poor treatment outcome and female gender, being HIV positive and being infected with moulds.

**Conclusion:**

Fungal infections play a significant role in SOM pathology in our setting. Diagnosis of fungal infections in developing countries should be improved so that appropriate management can be initiated on time to prevent associated complications.

## Background

Suppurative otitis media (SOM) is characterized by the inflammation of the middle ear and mastoid, tympanic membrane perforation as well as discharge [1]. The tympanic membrane perforation may result in increased exposure of the middle ear to pathogens [2, 3]. In developing countries, SOM is a major cause of preventable hearing loss [4–6], its incidence ranges from 7 to 46 % and is common amongst children of lower socio-economic status [7, 8]. In Tanzania, SOM constitutes a major cause of otorhinolaryngology clinic visits and contributes significantly to high morbidity and long term hearing loss [9–11]. While bacterial pathogens (most prominently *Pseudomonas aeruginosa* and *Staphylococcus aureus* [12–15]) have commonly been documented as the cause of SOM in developing countries, the role of fungal infections among patients with SOM is still underestimated [3, 16, 17]. Fungal infections among patients with SOM account for 2.1–25 % of cases [12–14] and *Aspergillus* spp*.* are the commonest cause accounting for 92.1 % of cases [12, 18].

Fungal infections are mainly attributed by compromised immune status, prolonged antibiotic use and immune suppressive therapy [19, 20]. This aspect of mycosis has gained importance over the recent years because of the excessive use of broad-spectrum antibiotics, and an increase in the number of immunodeficiency conditions such as immunoglobulin deficiency, malignant neoplasms, immunosuppressive therapy (corticosteroids and cytotoxic chemotherapy), diabetic mellitus as well as AIDS [3, 8, 21].

Fungi have been least documented as the cause of SOM in Africa probably due to lack of laboratory diagnose for fungal infections in these settings. Here, we determined the prevalence of fungal occurring in the otolaryngology clinic and surgical wards of Bugando Medical Centre (BMC), Mwanza Tanzania complicating cases of SOM.

## Methods

### Sample collection

A total of 410 patients with suppurative otitis media attending at BMC otorhinolaryngology clinic in a period of three months were investigated. For each patient with suppurative otitis media, ear swabs were aseptically collected using sterile cotton swabs (Heinz Herenz Hamburg, Germany).

### Microbiology

Swabs were transported to microbiology laboratory using Stuart transport media (HiMedia, India) and subjected to microscopic investigation by Gram’s stain as well as culture on sheep blood (BA), Mackonkey (MCA) and sabouraud’s dextrose agar supplemented with 50 mg/ml gentamicin and 50 mg/ml chloramphenicol (SDA) (Oxoid, UK). Plates were aerobically incubated at 35 °C for 24–48 h. Fungal cultures with at least 2+ growth were considered as significant [22]. Significant positive fungal pathogens on the SDA were further characterized while all other organisms were subjected to the normal procedures according to the BMC microbiology laboratory (Southern African Development Community Accreditation Service (SADCAS) with a unique number MED 002) standard operating procedures.

Growth on SDA plates was preliminary classified as mould or yeast based on the colony color and morphology. Yeast isolates were identified as growth of creamy to white colonies while moulds were identified as the filamentous colonies of various appearances [23]. CHROM agar (OXOID, England) was used as previously described for preliminary identifications of yeast in case of mixed growth [23].

Species identification was done by matrix-assisted laser desorption ionization-time of flight (MALDI-TOF) mass spectrometry (Bruker Daltonics, Bremen, Germany) on extracted cells harvested from agar plates (yeasts) or from overnight shaking cultures (moulds) in sabouraud’s broth (Oxoid, Wesel, Germany) as previous described [24, 25].

All moulds isolated were subjected to antimicrobial susceptibility testing using broth micro-dilution method according to EUCAST guidelines [26]. The antifungal agents tested were itraconazole, voriconazole (Discovery Fine Chemicals, Bournemouth, United Kingdom) and posaconazole (MSD Sharp and Dohme, Haar, Germany). Plates were incubated at 37 °C for 48 h and MIC values for all drugs were visually determined as the lowest concentrations with no visible growth.

For azole resistant *A. fumigatus* isolates, the *cyp51A* locus was amplified by PCR and the resulting fragments sequenced as described before [27].

All patients were managed according to the standard protocol at BMC. All patients were initially treated conservatively, including aural toilet and applications of appropriate antibiotics as per bacterial culture and sensitivity results [15]. Whenever fungal infections were suspected, patients were treated empirically; this included removal of fungal debris and application of boric acid or combinations of antibiotics and antifungals. In the current study, poor treatment outcome was defined as persistence of otorrhea while the treatment success was defined as the disappearance of the signs and symptoms after 14 weeks follow-up [15].

## Results

Out of 410 patients with SOM, 44 (10.7 %, 95 % CI 7.7–13.7) had significant fungal growth. The studied cases are summarized in Table 1. The median age (inter quartile range (IQR) of patients with fungal infections was 29.5 (IQR 16–43) years. Male formed majority of patients with fungal infections (*n* = 24, 54.6 %). The median duration of illness was 18 (IQR 5–24) weeks. Of the 44 patients with fungal infections, 6 (13.6 %) were HIV positive. On Mann Whitney ranksum test, there were non-significant difference in the median duration of illness between patients with fungal infections and those without fungal infections (18, IQR: 5–24 vs. 12 IQR: 5–24, *p* = 0.5749).

**Table 1:** Summary of 44 cases of SOM with fungal infection

ID	^a^Duration (weeks)	HIV	Isolates	Complication	Treatment outcome
ES025	60	Neg	*A. flavus*	Hearing loss	Not Cured
ES036	60	Neg	*A. flavus*	None	Cured
ES091	70	Neg	*A. flavus*	None	Cured
ES093	12	Neg	*A. flavus*	None	Cured
ES193	8	Neg	*A. flavus, A. versicolor*	None	Not Cured
ES026	4	Neg	*A. fumigatus*	None	Cured
ES028	32	Neg	*A. fumigatus*	None	Cured
ES095	20	Neg	*A. fumigatus*	None	Cured
ES027	90	Pos	*A. fumigatus, A. flavus*	None	Cured
ES057	18	Neg	*A. sydowii, P. chrysogenum*	Mastoditis	Cured
ES117	18	Neg	*A. versicolor*	None	Cured
ES130	32	Neg	*A. versicolor*	Hearing loss	Cured
ES143	18	Neg	*A. versicolor*	Hearing loss	Cured
ES179	18	Neg	A. versicolor	None	Cured
ES195	15	Neg	*A. versicolor, A. sydowii, P. sumatrense*	Hearing loss	Cured
ES112	24	Neg	*A. versicolor, P. citrinum*	None	Not Cured
ES001	24	Neg	*C. albicans*	None	Cured
ES002	12	Neg	*C. albicans*	None	Cured
ES016	2	Neg	*C. albicans*	None	Cured
ES030	12	Neg	*C. albicans*	None	Cured
ES032	48	Neg	*C. albicans*	Hearing loss	Not Cured
ES033	24	Neg	*C. albicans*	None	Cured
ES048	4	Neg	*C. albicans*	None	Cured
ES063	10	Neg	*C. albicans*	None	Cured
ES064	1	Neg	*C. albicans*	None	Cured
ES065	4	Pos	*C. albicans*	None	Cured
ES083	1	Neg	*C. albicans*	None	Cured
ES185	13	Neg	*C. albicans*	None	Cured
ES097	20	Neg	*C. albicans*	None	Cured
ES031	24	Neg	*C. parapsilosis*	Hearing loss	Not Cured
ES035	0.5	Neg	*C. parapsilosis*	None	Cured
ES061	6	Neg	*C. parapsilosis*	None	Cured
ES019	1	Pos	*C. tropicalis*	None	Cured
ES020	1	Pos	*C. tropicalis*	None	Cured
ES037	1	Neg	*C. tropicalis*	None	Cured
ES052	72	Neg	*C. tropicalis*	None	Cured
ES058	20	Neg	*C. tropicalis*	None	Cured
ES005	3	Neg	*C. tropicalis, A. fumigatus*	None	Cured
ES175	9	Pos	*P. chrysogenum*	None	Cured
ES077	18	Neg	*P. citrinum*	None	Cured
ES125	48	Pos	*P. citrinum*	None	Not Cured
ES085	6	Neg	*P. citrinum, A. versicolor*	None	Not Cured
ES132	24	Neg	*P. citrinum, P. lilacinus*	None	Cured
ES149	18	Neg	*P. sumatrense, A. versicolor*	None	Cured

^a^Duration indicate the period the patients has stayed with otitis media before presenting at otolaryngology clinic of BMC

Out of the 44 positive fungal cultures, 35 (79.6 %) produced pure growth of only one fungal species, while nine (20.4 %) had mixed fungal growth. A total of 23 (52.3 %) patients were positive for moulds and 21 (47.7 %) positive for yeast. *Candida albicans* was the most commonly isolated fungal (*n* = 13, 29.6 %) followed by *Aspergillus versicolor* (*n* = 8, 18.2 %) (Table1). A total of seven (15.9 %) patients had disease complication at time of enrollment; of them six (13.6 %) had hearing loss. On follow up, seven (15.9 %) had poor treatment outcome. Out of 366 patients with no fungal growth; 30 (8.2 %) had hearing loss as compared to 7/44 (15.9 %) of those with fungal infections (*p* = 0.0339).

All five strains of *Aspergillus fumigatus* isolates were resistant to itraconazole and showed reduced in vitro susceptibility to both voriconazole and posaconazole. Sequencing of the *cyp51A* locus revealed these isolates to carry the TR_34_/L98H resistance allele. *Penicillium citrinum* isolates were resistant to voriconazole and had reduced susceptibility to itraconazole and posaconazole*.* All *Penicillium sumatrense* were resistant to both itraconazole and voriconazole, and showed reduced susceptibility to posaconazole (Table 2).

**Table 2:** Antimicrobial susceptibility patterns

ID	Species	Itraconazole	Voriconazole	Posaconazole
ES93A	*Aspergillus flavus*	0.5	0.25	0.25
ES05B	*Aspergillus fumigatus*	32	1	0.5
ES026	*Aspergillus fumigatus*	32	4	1
ES95	*Aspergillus fumigatus*	32	2	1
ES95	*Aspergillus fumigatus*	32	1	0.5
ES95	*Aspergillus fumigatus*	32	2	1
ES57A	*Aspergillus sydowii*	0.5	0.5	0.25
ES95B	*Aspergillus sydowii*	0.5	0.25	0.5
ES85B	*Aspergillus versicolor*	0.25	0.03	0.25
ES93B	*Aspergillus versicolor*	0.13	0.5	0.5
M112A	*Aspergillus versicolor*	0.5	0.25	0.25
M117	*Aspergillus versicolor*	0.25	0.13	0.5
M130	*Aspergillus versicolor*	1	0.5	0.5
M143	*Aspergillus versicolor*	0.5	0.5	0.5
M149A	*Aspergillus versicolor*	0.5	0.25	0.25
M179	*Aspergillus versicolor*	0.06	0.03	0.06
M132B	*Paecilomyces lilacinus*	0.5	0.13	0.25
ES77	*Penicillium citrinum*	0.5	16	0.5
ES85A	*Penicillium citrinum*	0.25	8	0.25
ES95A	*Penicillium citrinum*	0.5	16	0.5
M112B	*Penicillium citrinum*	0.5	16	0.25
M125	*Penicillium citrinum*	0.5	16	0.25
M132A	*Penicillium citrinum*	0.5	16	0.25
ES95C	*Penicillium sumatrense*	32	32	1
M149B	*Penicillium sumatrense*	16	32	1
ES57B	*Penicillum chrysogenum*	0.13	0.13	0.13

There were non- significant association of the poor treatment outcome with increase in age, female gender, being HIV positive and being infected with moulds Table 3.

**Table 3:** Factors associated with poor treatment outcome among patients SOM due to fungal infection

Variable	Poor treatment outcome n (%)	OR(95 % CI)	P
Age^a^	29.5(IQR16-43)	1.04(1.00–1.08)	0.062
Sex			
Male (24)	3(12.50)	1	
Female (20)	4(20.00)	1.75(0.34–8.95)	0.5
HIV status			
Negative (38)	6(15.79)	1	
Positive (6)	1(16.67)	1.07(0.11–10.82)	0.95
Illness duration^a^	18(IQR 9–32)	0.98(0.94–1.02)	0.49
Fungal growth type			
Yeast (21)	2(9.52)	1	
Molds (23)	5(21.74)	2.64(0.45–15.36)	0.2

^a^Median

## Discussion

SOM is highly associated with lower social economic status, potentially due to poor hygiene, low access to medical care, or lack of knowledge [28]. In the current study, the majority of patients with positive fungal growth were from rural area where there are no hospitals with otolaryngology services. Most of these patients presented in BMC after six months of illness.

As seen in other studies [10], *C. albicans* and *Aspergillus* spp. were the most commonly isolated fungal from SOM specimens. The ability of *C. albicans* to maintain the synergistic relationship with bacterial pathogenic flora of skin like *S. aureus* and ecological niche of these isolates may explain the findings [29, 30].

Fungal have mainly been documented as opportunistic pathogens causing infections in immunocompromised patients. Among the 44 fungal-infected cases of SOM studied here, only six (15.9 %) were HIV positive, which increased the risk of getting poor treatment outcome of SOM by 1.2 fold. The immunocompromised state of these patients could explain this observation. However, this observation was not statistically significant. This necessitates the need to identify other risk factors associated with fungal infections among HIV-negative individuals in future studies.

In the current study, with eight isolates, *A. versicolor* was the predominant *Aspergillus* species. *A. versicolor* is a highly resilient/resistant fungus found in damp indoor environment, able to produce hepatotoxic and carcinogenic mycotoxin sterigmatocystin [31, 32].

Patients infected with moulds had 5.5 times higher risk of getting poor treatment outcome than patients with *Candida* infections. This could be explained by the fact that the majority (in fact all of the *A. fumigatus*, *P. sumatrense*, and *P. citrinum* isolates) were resistant to at least one commonly used antifungal agent. For the past two decades the increasing occurrence of *A. fumigatus* isolates resistant to common azoles antifungal agents has been described, including in Tanzania [33]. In our study, all *A. fumigatus* isolates indeed carried the TR_34_/L98H allele, which shows that the environmental occurrence of these isolates is also clinically relevant in Tanzania. Additionally, all isolates of *P. citrinum* and *P. sumatrense* were resistant to the lead antifungal agent itraconazole or showed reduced susceptibility to voriconazole. However in these species the underlying resistance mechanism is unknown.

In most clinical settings in resource-constrained countries like Tanzania where fungal diagnostics is underdeveloped; treatment of fungal infections relies solely on the empirical use of topical azole agents, which has previously been reported to be in effective for *A. versicolor* [34], the most frequent moulds isolated here and is probably similarly ineffective for azole-resistant *A. fumigatus*, a significant emerging problem.

In the current study 15.9 % of patients infected with fungi developed complications, mostly irrevocable hearing loss, proving that hearing loss is a major complication developed by patients with suppurative otitis media [35]. The clinical impact of fungal infections in patients with SOM should drive the effort to improve fungal diagnostics in developing countries.

One of the limitations of this study is the possibility of skin flora contamination during sample collections. However; the quantification of fungal growth significantly minimized the chances suggesting that majority of patients had real fungal infections.

## Conclusion

Fungal infections played a significant role in SOM pathology under low resource settings. Diagnosis of fungal infections in developing countries should be improved so that appropriate management can be initiated on time to prevent associated complications.

## Abbreviations

BMC: Bugando Medical Centre
CUHAS: Catholic University Health and Allied Sciences
EUCAST: European Committee on Antimicrobial Susceptibility Testing
HIV: Human Immunodeficiency Virus
IQR: Interquartile range
MALDI-TOF: Matrix-Assisted Laser Desorption Ionization-Time of Flight
SDA: Sabouraud’s Dextrose Agar
SOM: Suppurative Otitis Media
